# A cross-sectional survey of a public, evidence-based multimodal program for cognitive health in older adults

**DOI:** 10.1186/s13690-021-00670-9

**Published:** 2021-09-16

**Authors:** Barbara Studer-Luethi, Maria Brasser, Simon Lusti, Rahel Schaerli

**Affiliations:** 1 University of Bern, Bern, Switzerland; 2 University of Zürich, Zürich, Switzerland

**Keywords:** Cognitive health, Public prevention, Multimodal intervention, Cognitive stimulation

## Abstract

**Background:**

In recent decades, the proportion of older adults in the population has continued to rise, and with it, the need for intervention programs to maintain cognitive functions into old age. Multiple lifestyle factors, including physical, cognitive, and social activities, are crucial to forestalling a decline in cognitive functions. However, Covid-19 curtailed most activities, and therefore, strategies are needed to support older adults in remaining cognitively healthy. This study describes a newly developed and publicly available multimodal program, called “brain coach”, to support and stimulate cognitive activity in older adults. The autonomy supportive program integrates into daily life recommendations for evidence-based physical, cognitive, social, mindful, and creative activation exercises.

**Methods:**

The study design corresponds to a correlational, analytical, and cross-sectional study with 660 older adults, who participated in the program for at least 3 months and completed an online survey.

**Results:**

The survey results demonstrate that the average age of the participants was 71 years and 75 % were female. Participants experienced benefits in memory, well-being, attitudes towards the brain, and lifestyle habits. Importantly, time invested in the intervention and participant’s positive attitude toward brain health and neuroplasticity, show positive relationships with the experienced benefits.

**Conclusions:**

The results reveal the potential of a public program with a multimodal approach to increase cognitive health and promote an active lifestyle. Further research will explore the effects of such a multimodal intervention in a longitudinal randomized controlled trial study.

## Background

The ability to think, learn, and remember is essential to performing everyday activities and living independently. Therefore, as we age, one important goal is to maintain this general cognitive health. Because the proportion of older individuals in the general population continues to increase (United Nations, [64]), the demand for effective programs that support individuals in preventing cognitive decline is also growing. In addition, the adult brain structure has a great capacity to adapt and change in response to experience and training [19], so preventative approaches are promising.

Encouragingly, research findings from the previous two decades have shown that non-biological factors and lifestyle strongly influence cognitive decline [26]. That is, several lifestyle changes are crucial for cognitive health, including physical, cognitive, and social activities. Also, regulations imposed in response to Covid-19 have restricted various opportunities for cognitively stimulating activities, such as cultural events and other social interactions. Thus, interventions supporting and guiding older adults in their daily cognitive activities are even more important to protect them from cognitive decline. In addition, the advantages of web-based interventions became more obvious and technology acceptance increased. This is favorable, as a growing research field reveals the potential of technology and web-based interventions to enhance and enrich the lives of older adults. That is, use of technology can facilitate better interpersonal relationships, social connectedness, and physical health, which can be a substantial benefit for older adults ([1]; Chopik, [17]; Rogers & Mitzner, [53]).

The present investigation follows Williams and Camper’s (2010) appeal that “older adults may be advised to add new cognitive, physical, and social activities, and improved nutrition to support successful cognitive aging and to improve neuroplasticity.” That is, this study describes and investigates a public autonomy supportive online program for cognitive health by determining what kind of individuals participate in such a program, how much time they spend on the exercises, and whether they experience any benefits from the intervention. The implemented program offers support in maintaining cognitive health by sending registered individuals recommendations for evidence-based activities, lifestyle advice, and background information about the aging brain. The newly developed program emphasis the multimodality of effective prevention of cognitive decline by considering the evidence-based crucial lifestyle factors, which will be described in the following.

### Crucial lifestyle factors that prevent cognitive decline

A growing body of scientific research and the resulting World Health Organization (WHO) guidelines [4] have identified several crucial lifestyle factors that reduce the risk of cognitive decline and dementia.

The first factor is actively taking care of physical health by healthy food intake [46] and regular physical exercise [11]. individuals who are obese in midlife have a higher risk of dementia than those of healthy body weight. Indeed, both healthy food and physical exercise modulate the cellular and molecular processes in the brain vital for cognitive health, including optimizing growth factors such as brain-derived neurotrophic factors (e.g., [15]). In addition, intervention studies have shown that physical activity and optimized dietary behaviors increase cognitive functioning in older age. Increased physical activity is beneficial in protecting against cognitive decline [56].

The second factor is *cognitive activity*, such as lifelong learning and intellectual engagement. Higher cognitive activity in midlife and later life associates with delayed onset of cognitive impairment [66]. Cognitive interventions aim either to train memory and other cognitive processes directly or to train memory strategies that, in turn, improve cognitive performance [9]. Findings regarding the effects of cognitive interventions on the general cognitive functions of older adults are inconclusive (for a recent review, see [55]). Some cognitive interventions demonstrated significant benefits and indicated some transfer to daily life in healthy older adults [5, 9, 62] or older adults with mild cognitive impairments or dementia [73]. However, other cognitive interventions produced no significant benefits on the cognitive performance of older adults (e.g. [2]).

Another crucial factor is *social activity*. Few social contacts and low social have been associated with increased dementia risk [31]. Research has confirmed the importance of social connection through social activities to protect from cognitive decline and reduce feelings of isolation and loneliness (see [12, 42], for a meta-analysis). Various intervention studies have shown the positive effect of social activities on cognitive variables (e.g., [6, 18], for a meta-analysis). For example, a study of older adults with dementia revealed the positive influence of communication with family members and healthcare professionals on patients’ behavior and symptoms [67].

A range of other factors also influence cognitive function in older adults. One of them is *mindfulness,* which is associated with lower perceived stress and cortisol levels and improved well-being (cf. [25]). Because chronic stress affects memory and increases the risk of dementia [44], mindfulness-based interventions protect cognitive health (cf. [16]). In addition to this indirect effect on cognition through improved physiological mechanisms related to stress and immune function, a direct effect may occur through the repeated activation of attentional functions [39]. Indeed, evidence has shown that mindfulness-based interventions improve cognition in older adults with and without mild cognitive impairment [68, 74].

Another factor is *creativity*, linked to mental and physiological health indicators and cognition in older age (e.g., [58]). Individuals that frequently engage in highly creative activities demonstrate higher cognitive reserve than individuals with more routine activities [20]. Intervention studies with older adults with and without mild cognitive impairment or dementia found more significant positive effects on cognitive functions and daily living ability after creative therapy (e.g., drawing, dance, music, storytelling) than after standard cognitive training [41, 75]. In addition to interventions traditionally identified as creative, other creative activities include idea generation and improvising (cf. [22]).

Finally, *humor* leads to the release of endorphins in the brain and a reduction of cortisol, which produces psychological and physiological effects similar to the benefits of aerobic exercise (cf. [8]). Also, humor is a form of cognitive reappraisal, allowing individuals to reappraise daily stressors. Therefore, humor may decrease stress and protect cognitive abilities in older adults (cf. [40]). Humor interventions relieve chronic pain and enhance happiness and life satisfaction (e.g. [63]).

### A new autonomy-supportive multimodal program for cognitive health

“Brain coach” is a new public multimodal program that combines all the evidence-based factors mentioned above. It does so, because research on cognitive health suggests that the effect of these lifestyle factors on cognitive fitness may be additive and that a generally active lifestyle seems more effective than specific short-term interventions [32]. That is, single-component interventions often result in task-specific improvements with minimal, if any, generalization to overall cognitive functioning and daily life situations (e.g. [27, 52]). By comparison, multimodal interventions may produce more significant benefits by addressing multiple factors [14].

The present “brain coach” program reflects an autonomy-supportive intervention to increase autonomous motivation to invest in health behavior, which, based on the self-determination theory, can be reached by affording autonomy, relatedness, and competence of participants [54]. On this basis, the program aims to enable older adults and facilitate behavioral change through education, training, and relatedness (cf. [23, 60, 61, 76]). Also, the program aims to increase the awareness of brain health, because previous research has highlighted this need, as the general knowledge of dementia is inadequate [13, 24].

That is, the program informs participants about neuroplasticity and the impact of their lifestyle on their cognitive health (i.e., education). Participants receive weekly exercise recommendations for physical, cognitive, social, mindful, and creative activities for daily life (i.e., training). Participants can exchange and share their goals and experiences in a supervised online forum (i.e., relatedness).

### The present study

Whereas some existing lifestyle interventions combine two or three lifestyle factors, such as physical exercise and cognitive training [38, 48], little is known about effects of s multimodal program which include all the above-mentioned lifestyle factors. Also, there is only scarce evidence of personal characteristics that may influence cognitive intervention outcomes. One of them is stereotypes of older adults, such as the prevalent perception that they are cognitively impaired and forgetful (e.g., [71]). Such stereotypes were shown to negatively affect performance of older adults [33] and increase dementia worry [45]. Similarly, the belief in the brain’s capacity for neuroplasticity and a positive attitude towards the aging brain could positively impact outcomes of cognitive interventions.

Therefore, this article describes and investigates a new public multimodal program “brain coach” aiming at supporting older adults’ cognitive fitness and well-being and refining the attitude towards the aging brain. The cross-sectional survey follows several aims, including to determine:

1. The age and state of health of individuals who voluntarily participate in the public program and how much time participants spend on the exercises;
2. Whether participants experience any cognitive and wellbeing-related benefits from the intervention; and
3. Whether the program can potentially facilitate autonomously engaged cognitive activities of participants and a positive attitude towards the aging brain.

Based on the findings above, the authors assume that, through an autonomy-supportive intervention, participants will feel autonomously motivated to invest time in the weekly tasks. Through the background information and regular concrete advice, they will favorably change their attitude toward the aging brain, exert more cognitively stimulating activities in their daily lives, and experience these activities’ benefits.

## Methods

### Participants

The program began in the German-speaking part of Switzerland in March 2020. We conducted the cross-sectional survey from August to October 2020. A total of 660 participants completed the survey. Inclusion criteria were met when a participant used the intervention for personal reasons and exhibited an age minimum of 60 years, resulting in 542 participants included in statistical analyses. Demographic data comprised age (*M* = 71.47, *SD* = 6.34, range: 60–92), years of education (*M* = 13.80, *SD* = 1.82, range: 6–16), sex (76.2% female), civil status (3.0% unmarried, 45.0% married, 9.0% divorced, 9.0% widowed, and 1.5% civil union), and health condition (47.5% very good, 48.6% rather good, 3.7% rather bad, and 0.3% bad).

### Procedure

The study offered a free “brain coach” program advertised in the online media and newspaper articles. Individuals registered for the intervention received a sheet with exercises and background information by email every week or every second week (length: approx. 2–5 pages). Participants were encouraged to implement the exercises and activities in their daily lives and exchange with other participants in an online discussion forum. They were free to choose which of the exercises they performed, when, and how intensely. After 5 months, the online survey was unannouncedly sent by email to individuals who participated in the program for at least 3 months (*n* = around 1500 individuals) at the date of the evaluation. Participants gave consent to participate in the study as they filled out the survey. Thus, the survey was completely voluntary and participation anonymous.

### Program

Dr. Barbara Studer from the Competence Centre for Learning and Memory at the University of Bern developed the “brain coach” program in collaboration with professionals from the University of Zurich. The program takes into account findings from various applied studies that examined the effects of cognitive, emotional, social, and motivational interventions. The purpose of the program is to encourage and instruct participants to make particular activities and specific exercises part of their daily/weekly activities. Notably, the program offers exercises and background information to participants in the sense of an exercise buffet, where participants chose which tasks to execute. In addition, the authors applied self-determination theory for adult learning (i.e., autonomy, competence, and relatedness) to offer interventions supportive of individual autonomy [21]. The program consists of three parts, a collection of beneficial exercises to increase training and enablement, psychoeducative information (education), and a supervised online discussion forum (relatedness). An example of an English version of a “brain coach” exercise sheet is linked here: [https://cdn1.site-media.eu/images/document/5399492/BraincoachProgram.pdf].

#### Part one

Weekly task sheet (3–6 pages) containing exercise descriptions and links to short video demonstrations of various cognitive activation exercises for everyday life. The task units are of minimal investment, approximately 5–10 min each. Thus, the participants could perform them during their daily routine. The evidence-based exercises are designed to support cognitive health by addressing five areas: memory through cognitive training tasks and memory strategies such as N-back exercises and the loci method; coordination through physical coordination tasks such as balance and juggling exercises; mindfulness through exercises for increased self-awareness such as a breathing task; creativity through generating creative new ideas such as finding new applications for an item; community through ideas for social activities without physical contact due to Covid-19; and humour through funny jokes and cartoons.

#### Part two

The task sheets includes psychoeducational information about brain functions and neuroplasticity. The explanations focus on the effects of the exercises and strategies on neural processes and cognitive health. Examples of topics: “Use it or lose it”, how physical exercise changes our brain, lifelong learning, strategies to change habits on our own [30]. Importantly, these oral or written psychoeducative impulses are in simple words, so that little prior knowledge was needed to understand the messages.

#### Part three

A neuropsychologist supervised an online discussion forum. Participants were encouraged to exchange experiences, failures, and successes and ask questions answered by the neuropsychologist in the forum.

### Self-reported measures

#### Characteristics of participants

##### Personality

The Big Five Inventory, a 10-item questionnaire that records responses on 5-point Likert scales [51], measures personality traits.

##### Attitude towards the aging brain

The study assessed participants’ attitudes toward their aging brains with a five-question survey designed by the authors. One question assessed general belief in neuroplasticity, and the other four questions assessed the strength of positive and negative attitudes towards the aging brain on Likert scales ranging from 1 (no, not at all) to 4 (yes, a lot):

##### Belief in neuroplasticity

Participants’ belief in their aging brains’ plasticity was assessed with the question, “What do you think about your aging brain?” Participants recorded responses on an open scale ranging from 0 (i.e., age-dependent cognitive decline) to 10 (i.e., the brain stays adaptive and capable).

##### Positive attitude

The authors calculated the strength of positive attitude (i.e., confidence in conserved learning ability) from the mean value for the general conviction about the brain’s plasticity and personal confidence in influencing cognitive fitness positively.

##### Negative attitude

The authors calculated the strength of negative attitude (i.e., worry about cognitive decline) from the mean value of general worries about biological, age-dependent cognitive decline and individual concerns about a decline in learning and memory.

#### Intervention commitment

The study assessed the *frequency* with which participants performed individual exercises with the question “How regularly do you perform exercises from the “brain coach” program on average?“ Five answer options were available, ranging from “less than every other week“ to “daily.“ The authors assessed *weekly time* invested in the exercises with the question, “How much time do you spend weekly on average doing exercises from the “brain coach” program?“ The five answer options ranged from “less than 15 min a week” to “more than 2 h a week.” Finally, the authors calculated the *overall time invested for the intervention* by multiplying the time invested in the intervention per week by the number of weeks since the participant’s registration date.

#### Perceived intervention-induced benefits

##### Benefit in cognitive fitness

The study asked participants about the perceived effect of the intervention on their memory performance (“Have you experienced any benefit from the “brain coach” exercises for your cognitive fitness, such as memory?”).

##### Benefit in well-being

The study asked participants about the perceived effect of the intervention on their well-being (e.g., “Have you experienced any benefit from the “brain coach” exercises for your well-being, such as mindfulness, so far?”). -.

##### Benefit from brain health-related attitude

The study asked participants whether the intervention changed their attitude toward their brain health (e.g., “Have the “brain coach” exercises changed your attitude towards your brain health?”)

##### Benefit from activities

The study asked participants whether the intervention changed their behavior (e.g., “Have you changed any activities that benefit the brain or body in everyday life because of the “brain coach” program?’). Participants reported their responses on a Likert scale ranging from 1 (no) to 4 (yes, a lot).

### Data analysis

Descriptive statistical analyses were performed as mean and standard deviation and as frequency distribution for the sample characteristics, intervention commitment, and self-reported benefit of the intervention. In addition, the authors performed correlation analyses to analyze the relationship between participants’ characteristics (belief in neuroplasticity, attitude towards brain health, personality), intervention commitment (total time investment), and self-reported benefits through the intervention (change in memory, mindfulness, attitude, activities). Kendall-Tau-b and Spearman’s correlation coefficients were used, but only results from the former are reported here for clarity. Finally, the authors performed an analysis of multiple linear regressions to analyze the variance and the strength of the relationship between investment, attitude, and experienced profit. The dependent variable was a generalized experienced benefit, defined by the average of experienced effects on cognitive fitness and well-being and brain-health-related attitude and behavior. Two independent variables were time investment and belief in neuroplasticity.

## Results

**Fig. 1:**
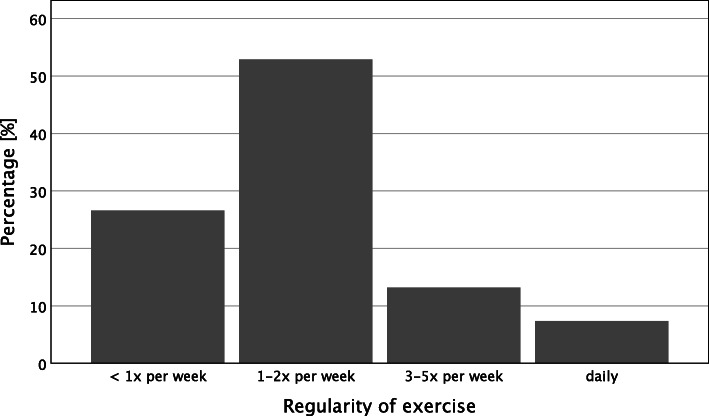
Regularity of exercise on a temporal scale

**Fig. 2:**
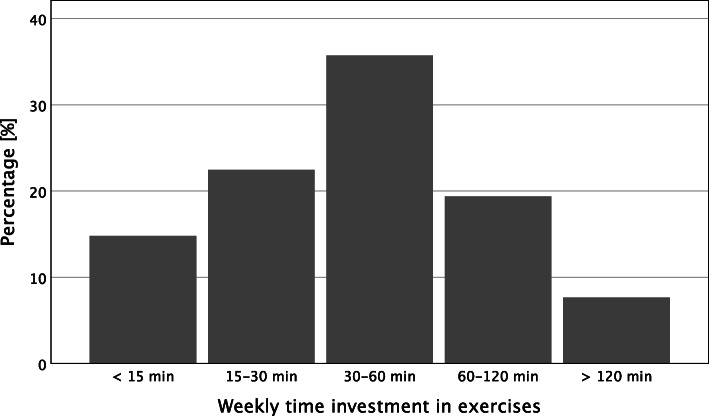
Weekly time investment in exercise

**Fig. 3:**
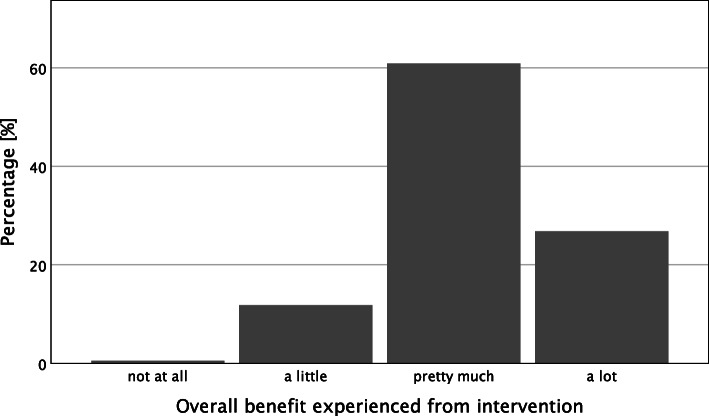
Overall benefit experienced from intervention represented in verbal categories

Descriptive results and frequency distributions of sample characteristics, intervention commitment, and self-reported benefit appear in Table 1 and Figs. 1, 2 and 3. In addition, correlations between these variables appear in Table 1.

**Table 1:** Descriptive statistics and correlations for benefits experienced, time investment, attitudes, and big five personality traits

Variables	*M*	*SD*	1	2	3	4	5	6	7	8	9	10	11	12	13
*Benefits experienced*															
1. Cognitive Fitness	3.01	0.61	-												
2. Well-being	3.05	0.75	.43**	-											
3. Attitude	3.14	0.78	.34**	.31**	-										
4. Activity	2.93	0.81	.40**	.40**	.42**	-									
*Time investment*															
5. Time Investment	22.31	28.13	.26**	.26**	.20**	.29**	-								
*Attitude towards brain*															
6. Belief in Neuroplasticity	7.58	2.00	.18**	.14**	.15**	.10*	.23**								
7. Positive Attitude	3.24	0.57	.15**	.13**	.16**	.27**	.17**	.07^n.s.^	-						
8. Negative Attitude	2.45	0.74	-.02^n.s.^	.00^n.s.^	-.06^n.s.^	.04^n.s.^	-.06^n.s.^	-.41**	.25**	-					
*Personality traits*															
9. Consciousness	4.00	0.73	.05^n.s.^	.04^n.s.^	.06^n.s.^	.06^n.s.^	.14**	.19**	.06^n.s.^	-.16**	-				
10. Agreeableness	3.67	0.69	.05^n.s.^	.14**	.06^n.s.^	.06^n.s.^	.12**	.14**	.08^n.s.^	-.04^n.s.^	.09*	-			
11. Neuroticism	2.71	0.87	-.09*	-.06^n.s.^	-.08^n.s.^	-.07^n.s.^	-.10*	-.20**	.02^n.s.^	.12**	-.11**	-.21**	-		
12. Extraversion	3.26	.98	.11*	.08^n.s.^	.06^n.s.^	.08^n.s.^	.09*	.23**	.09*	-.12**	.16**	.11**	-.16**	-	
13. Openness	3.75	0.89	-.02^n.s.^	.10*	.11*	.06^n.s.^	.09*	.15**	.14**	-.08*	.11**	.12**	-.11**	.15**	-

*n* sample size, *M* mean, *SD* standard deviation

^*n.s.*^*p* > 0.05. * *p* < 0.05. ** *p* < 0.01

### Time investment

On average, participants used the intervention twice per week (Fig. 1). The average of hours invested related to the intervention was 22.31 (*SD* = 28.13), and the mean of weekly invested hours related to the intervention was 0.65 (*SD* = 0.72; Fig. 2). The average days of participation were 135.04 (*SD* = 36.75).

### Experienced benefit

Participants described the experienced benefit of intervention as “pretty much” on the four-point Likert Scale (*M* = 3.04, *SD* = 0.56) with the highest benefit on the attitude towards brain health (*M* = 3.14, *SD* = 0.78; Fig. 3).

### Time investment is related to benefit

There was a significant relationship between time invested and benefit experienced in cognitive fitness, *r =* .26, *p* < .01; well-being, *r* = .26, *p* < .01; attitude, *r* = .20, *p* < .01; and activity, *r* = .29, *p* < .01 (Table 1).

### Attitude toward the aging brain is related to benefit

The belief in neuroplasticity was significantly related to benefit experienced in cognitive fitness, *r =* .18, *p* < .01; well-being, *r* = .14, *p* < .01; attitude, *r* = .15, *p* < .01; activity, *r* = .10, *p* < .05; and time investment, *r* = .23, *p* < .01 (Fig. 1). The regression analyses, with experienced general benefit as a dependent variable and time invested and belief in neuroplasticity as independent variables, showed a significant regression eq. (F (2,357) = 34.737, *p* < .000), with an *R2* of .093. Furthermore, participants’ general experienced benefit increased by 0.01 rating points for each hour of investment and by 0.136 for each rating point of belief in neuroplasticity. Thus, both time investment and belief in neuroplasticity were significant predictors of generalized benefit experienced.

## Discussion

To our knowledge, this is the first study that investigates a multimodal public online program to foster cognitive activation and health behavior of older adults. It is of considerable length and tailored to life circumstances that have changed in the Covid-19 pandemic. The “brain coach” program applies the conceptual framework that cognitive health is positively related to an active lifestyle that integrates physical, cognitive, and social activities. It is an autonomy-supportive program that aims to enable older adults by increasing their autonomous motivation and competence through education, training, and relatedness. Participants receive information about how everybody can influence their brain health and choose cognitively stimulating activities and exercises. They also receive messages encouraging them to make these choices part of their daily routine and exchange their experiences with others. This cross-sectional study reveals the characteristics of the participants, the degree of commitment to the offered program, and the benefit experienced from it: Mean age of the participants was 71 years, 75 % were female, they invested 30–60 min/week in the activities of the program, and they reported experienced benefits in cognitive fitness, well-being, and attitude towards their brain, and more cognitive activities in their daily life. These benefits showed positive relationships with time invested in the intervention and participant’s positive attitude toward brain health and neuroplasticity.

### Characteristics of participants

The mean age of participants was 71 years. This age is in line with the finding that older people’s worries about their memory and dementia are highest around age 70 (see [10]). However, a recent review found inconclusive results about the influence of age on worries about dementia [70]. In addition, three-quarters of the participants were female. The gender distribution is comparable with memory and health intervention studies, where 79% or even 82% of at-risk older participants were female [43, 57]. Other findings confirm that women are more concerned about their health and about developing dementia, engage in more health-promoting behaviors, and seek preventive healthcare measures more often (see [50], for a review). Also, the findings align with prior research showing that women tend to be more likely than men to prefer unsupervised activities and activities at unspecified time [65]. Several explanations could account for the gender differences. These include women’s greater willingness and ability to take care of themselves when they are sick, several stereotypical masculine behaviors, and men’s inclination to self-reliance, physical toughness, and emotional control (cf. [50]). However, there is no consensus regarding whether men or women benefit more from interventions (see [7, 59], for reviews). Our sample showed no difference between males and females in intervention-related benefits (*t*(367) = ;0.70, *p* > .05.).

More than 90% of the participants were in good or excellent health. Good health is not surprising because the intervention was noncommittal and online and not restricted, for instance, to a health care facility. Consequently, only individuals with access to the internet and sufficient mental and self-regulation resources to implement the exercises independently could participate. In addition, other research has demonstrated that perceived health status influences participation in health promotion behavior [57].

### Intervention commitment

The average weekly time invested in exercises was around 50 min, performed on average twice per week. This time investment suggests that intervention commitment was relatively high and confirms the animating effect of the intervention. It is also in line with other interventions in older adults, which elicited between one and three home exercise sessions per week on average (cf. [49]). Finally, it corresponds to the suggested intensity of lifestyle intervention activities [29].

### Self-reported perceived benefits and time investment

Generally, temporal change presents a significant stressor for older adults’ well-being and may have affected participants during the time frame of the intervention. For example, the stress associated with the COVID-19 lockdown (in Switzerland from March to April 2020) restricted movement and created social isolation. In addition, as a corresponding survey shows, the global pandemic had some adverse effects on older adults’ emotional well-being and loneliness ([37]).

Nevertheless, around 60% of the participants estimated that they benefitted substantially from the intervention, and more than 25% reported very high benefits. Because this survey was cross-sectional, retrospective bias cannot be excluded, and causal interpretations cannot be made. Nevertheless, the result suggests that participants experienced positive effects from the exercises introduced in the intervention. The evidence that supports this claim is the finding that self-reported benefits related closely to the time invested in intervention-related exercises. This finding confirms that the higher the inactive investment lifestyle, the higher the benefits experienced over time (cf. [32]).

Finally, participants experienced comparable positive effects on their memory performance, well-being, attitude towards their brain health, and activities in daily life. This finding confirms that interventions integrating recommendations and instructions of evidence-based exercises and lifestyle activities for cognitive health exert a positive effect on cognition and also on well-being, and lifestyle activities (e.g. [27, 29, 47]). The participant’s experience of positive change in attitude towards their brain is in line with findings suggesting that brief exposure to aging stereotype content can change the levels of dementia worry ([45]). These findings indicate that addressing aging stereotypes (e.g. forgetfulness) and informing about neuroplasticity may be one way of increasing positive attitude towards cognitive health and decreasing dementia worry.

### Relationship between attitude towards brain health and intervention outcomes

Individuals with a stronger belief in neuroplasticity indicated higher time investment in the intervention-related exercises and reported higher benefits experienced in cognitive fitness, well-being, attitude, and activity. This finding corroborates studies demonstrating that individuals with positive age stereotypes show less and slower cognitive decline than individuals with negative stereotypes [35, 36]. Thus, a positive attitude to brain health may promote a willingness and motivation to invest in healthy behavior and protect cognitive health.

### Limitations and outlook

The main limitation of this study is the cross-sectional method, which precludes any conclusions about causalities and intervention effects (cf. [28]). Also, all measures were self-reported and therefore prone to bias and social desirability [72]. Furthermore, we do not have any information about the individuals who participated in the program but did not follow our call to complete the survey. Therefore, we do not know if there is any systematic difference between persons who may have left the study or who continued participation but did not fill out the questionnaire instead of those who continued participation and filled out the questionnaire. It is possible that individuals not participating in the survey could be more inactive or could have experienced fewer effects of the intervention. Finally, the external validity of the findings may be low because the study occurred online, within a specific context of a developed country in Western Europe, and most participants describe their health as “rather good” or “good”. The results may not be generalizable to participants with lower health or socio-ecological context or from countries that may not have access to internet-based interventions or other resources (cf., [3]).

Future research should conduct more longitudinal randomized control trials with large samples of subjects to evaluate the effects of lifestyle interventions, applied at different stages of life, on cognitive performance, well-being, as well as real-life changes.

## Conclusions and implications

The cross-sectional survey of this autonomy supportive multimodal “brain coach” program demonstrates that participants seem to be autonomously motivated to execute the exercises, weekly invest more than 30 min for it, and profit from it. That is, the public program animated healthy older adults, primarily female, to invest in their cognitive health and to favorably change their attitude towards the aging brain. Participants experienced the resulting benefits in their daily lives through increased cognitive performance and well-being. The results reveal the potential of a multimodal approach, which integrates five instead of only two areas, as implemented in previous studies (e.g., cognitive and physical exercise; [34], to increase cognitive abilities, well-being, attitude towards the brain, and stimulating activities in daily life. That is, public lifestyle interventions should effectively communicate scientific evidence about the impact of an active lifestyle with physical, cognitive, social, mindful, and creative activities, on preventing cognitive decline.

Older adults seem to appreciate and profit from the autonomy supportive opportunity to choose a preferred exercise from an offered selection of activities and beneficially integrate these into their daily lives. This finding aligns with prior research that the perceived autonomy is crucial for older people, whose ability lessens with age, yet perceived autonomy increased their quality of life and satisfaction with daily routines [69]. However, this intervention appears to address women more than men. More research is needed to determine how to motivate men to invest in their cognitive health and tailor interventions to meet gender-specific interests.

Considering that the identification of factors increasing the effects of cognitive prevention programs in older adults is a prerequisite for successful prevention, our findings may have implications for implementation of such programs. That is, our findings highlight the importance of participants’ attitudes toward brain health to their investment in behavior that protects cognitive health. The attitude towards the aging brain could be assessed prior to an intervention to tailor transferred information about the aging brain. Moreover, it seems necessary at a societal level to find ways to promote positive age stereotypes and attitudes towards the aging brain. Neuropsychoeducative information, such as about the neuroplasticity of the aging brain, should always be part of cognitive intervention programs. Recommendations could be personalized to participants’ lifestyles and attitudes towards brain health to maximize their utility (cf. [7]). Furthermore, lifestyle interventions should occur early as possible to protect older adults’ brains before impairment occurs.

In sum, our study indicates that an autonomy supportive lifestyle intervention for cognitive health which offer a choice of evidence-based exercises and activities can increase the frequency of cognitively stimulating activities executed in daily life and the experienced cognitive fitness and well-being. Increasing individual’s awareness of the brain’s capacity for plasticity and the resulting importance of cognitive stimulation may motivate people to adopt and maintain a cognitively active lifestyle.

## Data Availability

The datasets used and analysed during the current study are available from the corresponding author on reasonable request.

## Abbreviations

Covid-19: Coronavirus disease of 2019
WHO: World Health Organization
